# Exploring Digital Maze Performance Features Associated with PET Spatial Extent (EXT) in Cognitively Normal Older Adults

**DOI:** 10.1002/alz70861_108811

**Published:** 2025-12-23

**Authors:** Grace A Del Carmen Montenegro, Talia L. Robinson, Eva N Pintucci, Jessie Fanglu Fu, Jackson C Thompson, Michelle E. Farrell, Michael J. Properzi, Dana Penney, Randall Davis, Reisa A. Sperling, Keith A. Johnson, Rebecca E. Amariglio, Dorene M. Rentz

**Affiliations:** ^1^ Massachusetts General Hospital, Harvard Medical School, Boston, MA USA; ^2^ Massachusetts General Hospital, Boston, MA USA; ^3^ Brigham and Women's Hospital, Boston, MA USA; ^4^ Athinoula A Martinos Center for Biomedical Imaging, Massachusetts General Hospital, Harvard Medical School, Charlestown, MA USA; ^5^ Department of Neurology, Massachusetts General Hospital, Harvard Medical School, Boston, MA USA; ^6^ Lahey Hospital and Medical Center, Lexington, MA USA; ^7^ MIT Computer Science And Artificial Intelligence Laboratory, Cambridge, MA USA

## Abstract

**Background:**

The digital maze test (dMaze) may serve as a sensitive tool to detect cognitive changes in preclinical Alzheimer’s disease (AD). In prior work with a largely cognitively normal (CN) sample, we showed specific dMaze features such as pen stroke count and Pen Off Page Time, and a pattern of faster speed with greater backtracking and wall penetration errors are associated with global amyloid burden. Here, we sought to further evaluate the sensitivity of dMaze in preclinical AD by evaluating its association to a novel measure of amyloid spread/extent (EXT), designed to capture earlier stages of amyloid deposition.

**Method:**

A cohort of 123 (CN) older adults completed the dMaze test and [11C]Pittsburgh Compound B (PiB, DVR) PET. Maze features were extracted from “no‐choice” (NC; simple path following with no decision points) and “choice” conditions (CH; containing decision points). Participants were categorized into three EXT groups based on EXT%: EXT− (<7.2%, no amyloid, n=73), EXT+ (7.2–95.6%, evidence of amyloid spread, n=32), and EXT++ (>95.6%, widespread amyloid, n=25), based on percent of cortical voxels exceeding the PiB positivity thresholds. Linear regression models assessed the relationship between each dMaze feature and EXT group, adjusting for age, sex, and education. dMaze features were further analyzed in a subsample of EXT+ participants only, using EXT% as a continuous predictor.

**Result:**

In the CH condition, the EXT++ group exhibited significantly longer Pen Off Page Time (β=4.70, *p* =0.03, R²=0.13) and higher speed variability (β=1.69, *p* =0.01, R²=0.19) than the EXT− group. Follow‐up analyses restricted to EXT+ participants only, showed that higher EXT% was significantly associated with greater speed (β=0.28, *p* =0.03, R²=0.19) and Wall Penetration Count (β=0.03, *p* =0.03, R²=0.18) in the NC condition.

**Conclusion:**

Our findings demonstrate that dMaze features can detect subtle cognitive deficits associated with early amyloid spread even in a CN only sample. While specific CH maze features differentiated individuals without amyloid and with evidence of amyloid spread, unique features on the NC maze were associated with the level of spread within a subthreshold group (EXT+). These results build on work with global amyloid burden by showing that dMaze is sensitive to subtle and nuanced deficits in executive function even in preclinical AD.

